# Calcium in Kenyon Cell Somata as a Substrate for an Olfactory Sensory Memory in *Drosophila*

**DOI:** 10.3389/fncel.2018.00128

**Published:** 2018-05-14

**Authors:** Alja Lüdke, Georg Raiser, Johannes Nehrkorn, Andreas V. M. Herz, C. Giovanni Galizia, Paul Szyszka

**Affiliations:** ^1^Department of Biology, Neurobiology, University of Konstanz, Konstanz, Germany; ^2^International Max Planck Research School for Organismal Biology, Konstanz, Germany; ^3^Fakultät für Biologie, Ludwig-Maximilians-Universität München, Martinsried, Germany; ^4^Bernstein Center for Computational Neuroscience, Munich, Germany

**Keywords:** *Drosophila melanogaster*, olfaction, sensory memory, mushroom body, Kenyon cells, trace conditioning, calcium imaging

## Abstract

Animals can form associations between temporally separated stimuli. To do so, the nervous system has to retain a neural representation of the first stimulus until the second stimulus appears. The neural substrate of such sensory stimulus memories is unknown. Here, we search for a sensory odor memory in the insect olfactory system and characterize odorant-evoked Ca^2+^ activity at three consecutive layers of the olfactory system in *Drosophila*: in olfactory receptor neurons (ORNs) and projection neurons (PNs) in the antennal lobe, and in Kenyon cells (KCs) in the mushroom body. We show that the post-stimulus responses in ORN axons, PN dendrites, PN somata, and KC dendrites are odor-specific, but they are not predictive of the chemical identity of past olfactory stimuli. However, the post-stimulus responses in KC somata carry information about the identity of previous olfactory stimuli. These findings show that the Ca^2+^ dynamics in KC somata could encode a sensory memory of odorant identity and thus might serve as a basis for associations between temporally separated stimuli.

## Introduction

Odorants evoke odorant-specific spiking patterns across olfactory receptor neurons (ORNs), which drive odorant-specific neural activity patterns in different brain areas. In mammals, odorants evoke activity first across ORNs in the olfactory epithelium and then in glomeruli in the olfactory bulb, followed by responses in the olfactory cortex, the amygdala, and other brain areas (Uchida et al., 2014). Similarly, in insects, activity across ORNs first drives responses in olfactory glomeruli in the antennal lobe, and later in higher brain regions such as the mushroom bodies (Galizia, 2014). In neurons, activity (e.g., membrane potential or changes in cytosolic Ca^2+^ concentration) in the dendrite is often different from activity in axon terminals. Therefore, in order to understand information processing along neural signaling chains, it is necessary to record neural activity in subcellular compartments. Ca^2+^ imaging is a suitable technique: Ca^2+^ enters the cytosol through ligand-gated and voltage-dependent Ca^2+^ channels (as a function of local membrane potential) and/or through release from intracellular stores (as a function of second messenger cascades, including Ca^2+^ itself), and its concentration is restored through buffers, membrane pumps, and carriers (Augustine et al., 2003). Ca^2+^ has multiple functions: in presynaptic terminals Ca^2+^ triggers vesicle release (Stanley, 1997); at post-synaptic sites Ca^2+^ is involved in long-term synaptic plasticity (Khodakhah and Armstrong, 1997); in somata, Ca^2+^ influences gene transcription (Lyons and West, 2011). Therefore, recording the dynamics of cytosolic Ca^2+^ across neuronal compartments sheds light on different cellular processes.

**FIGURE 1:**
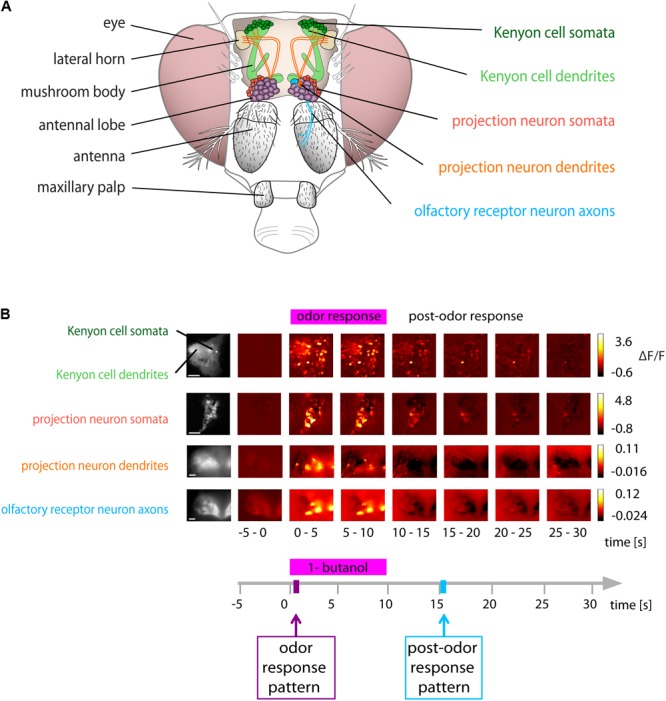
Odor and post-odor responses along the olfactory pathway. **(A)** Schematic of the olfactory system of *Drosophila*. (Left): gross anatomy. (Right): areas investigated in this study. **(B)** Spatial distribution of Ca^2+^ activity during and after olfactory stimulation (here with 1-butanol) in the neuronal compartments along the olfactory pathway. Color coded ΔF/F images show the average of 5 s recording time (i.e., 25 frames). All areas showed distinct responses upon odorant stimulation. (Scale bars 20 μm). See also calcium imaging movies on http://neuro.uni-konstanz.de/luedke/.

The olfactory system of flies consists of several layers of neurons (**Figure 1A**). ORNs that express the same receptor type coalesce in the same glomerulus (Vosshall et al., 2000; Couto et al., 2005). Within a glomerulus, all ORNs converge onto uniglomerular projection neurons (PNs) (Kazama and Wilson, 2009). Excitatory and inhibitory local neurons interconnect glomeruli and mediate gain control (Olsen et al., 2007; Silbering and Galizia, 2007; Root et al., 2008) and decorrelate odorant-evoked activity patterns (Wilson et al., 2004). Lateral inhibition via local neurons and short-term synaptic depression at the ORN-PN synapse shorten PN’s odorant-evoked responses, increase their sensitivity for concentration changes (Wilson et al., 2004; Bhandawat et al., 2007), and increase their odorant-specificity (Olsen and Wilson, 2008; Silbering et al., 2008). PNs connect the antennal lobe with the mushroom body calyx (**Figure 1A**). The mushroom body-intrinsic Kenyon cells (KCs) respond to fewer odorants and generate fewer spikes than individual PNs (Perez-Orive, 2002; Szyszka et al., 2005; Turner et al., 2008). This sparsening of KC responses is caused by the divergent connectivity between PNs and KCs (Jortner et al., 2007; Caron et al., 2013; Eichler et al., 2017) and by inhibitory feedback (Demmer and Kloppenburg, 2009; Lei et al., 2013; Lin et al., 2014). KCs send their axons to the mushroom body lobes, where they receive input from dopaminergic neurons (Aso et al., 2010; Burke et al., 2012). In standard classical conditioning, the simultaneous presentation of an odorant and a reward or punishment (reinforcer, encoded by dopaminergic neurons) induces an associative odor memory in flies (Tully and Quinn, 1985), which is encoded as a change in synaptic strength between KC axon terminals and mushroom body output neurons. The formation of this memory depends on cytosolic Ca^2+^ signaling and requires coincidence detection of an elevated Ca^2+^ concentration in KC axon terminals (for the odorant) and activated dopamine receptors (for the reinforcer) by an adenylyl cyclase (McGuire, 2001; Schwaerzel et al., 2002; Tomchik and Davis, 2009; Gervasi et al., 2010; Séjourné et al., 2011; Aso et al., 2014; Cohn et al., 2015; Hige et al., 2015; Owald et al., 2015).

However, this model of associative odor learning cannot explain associative odor learning during trace conditioning. In olfactory trace conditioning, the reinforcer arrives after the odor stimulus has already terminated, and there is no overlap between the two stimuli. In this situation, the reinforcer arrives when the cytosolic Ca^2+^ in KC axon terminals is already back to baseline (Galili et al., 2011; Shuai et al., 2011; Szyszka et al., 2011; Dylla et al., 2013, 2017). Where, then, is coincidence detection possible for associative trace conditioning? Trace conditioning requires an as-yet elusive sensory memory of the odorant. Such sensory odor memory is a form of short-term, non-associative memory, which may also be used in other contexts: honey bees use a sensory odor memory to solve delayed-matching-to-sample tasks (Giurfa et al., 2001), and flies and moths use a sensory odor memory to continue their odor source search after losing an attractive odor plume (Vickers, 2005; van Breugel and Dickinson, 2014; Saxena et al., 2017). Behavioral data show that these sensory odor memories can last for several seconds. Previous studies have proposed that such sensory odor memories could be encoded by prolonged changes in Ca^2+^ concentrations (Tomchik and Davis, 2009; Galili et al., 2011; Szyszka et al., 2011; Yarali et al., 2012) or synaptic modification (Drew and Abbott, 2006; Izhikevich, 2007; Cassenaer and Laurent, 2012), but the cellular localization of sensory odor memories is still unknown.

Because in *Drosophila*, associative odor learning requires coincidence detection of an elevated cytosolic Ca^2+^ concentration (representing the odorant) and activated dopamine receptors (representing the reinforcer) (Tomchik and Davis, 2009; Gervasi et al., 2010), we here characterized cytosolic Ca^2+^ signals across neurons and neuronal compartments in the olfactory system of *Drosophila*. We used the genetically encoded Ca^2+^ reporter GCaMP to record odorant-evoked cytosolic Ca^2+^ signals in ORN axon terminals (in antennal lobe glomeruli), two compartments of PNs (dendrites and somata in the antennal lobes), and two compartments of KCs (dendrites and somata in the mushroom bodies). We focused on how odorant-evoked Ca^2+^ signals develop during the presence of odorants and after their offset in experimentally naïve, unconditioned flies. In particular, we tested whether stimulus-outlasting Ca^2+^ signals could encode sensory odor memories. We found that all neuronal compartments show odorant-specific Ca^2+^ activity patterns during the stimulus time. Furthermore, all compartments showed prolonged odorant-evoked Ca^2+^ concentration changes after stimulus offset. However, only the somata of KCs showed Ca^2+^ signals with a pattern that could encode odorant identity for several seconds after stimulus offset. Therefore, prolonged Ca^2+^ signals in KC somata encode a sensory odor memory which could be used to associate an odorant with a delayed reinforcer during trace conditioning.

## Materials and Methods

### Nomenclature

To avoid terminological confusion, we define the terms used in this paper here. *Classical conditioning*: pairing a conditioned stimulus with a reinforcer. *Standard conditioning* (synonym: delay conditioning): the most frequent form of classical conditioning with overlapping conditioned stimulus and the reinforcer. *Trace conditioning*: classical conditioning with a temporal gap between the offset of the conditioned stimulus and the onset of the reinforcer. Trace conditioning requires a *sensory odor memory* (synonym: trace). A sensory odor memory differs from a working memory, as a sensory odor memory does not require attention.

### Flies

**FIGURE 2:**
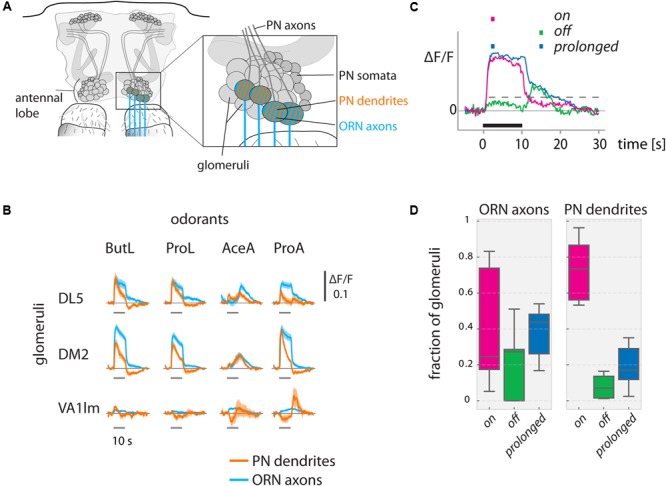
Odorant-evoked activity in ORN axons and PN dendrites in the antennal lobe. **(A)** Schematic of the antennal lobe. ORN axons (blue) and PN dendrites (orange) were measured in olfactory glomeruli. **(B)** Ca^2+^ responses show odorant-specific dynamics. Ca^2+^ responses (ΔF/F) are shown for ORN axons (Orco-Gal4, blue) and PN dendrites (GH146-Gal4, orange) for exemplary glomeruli. Odorant stimuli were 10 s long. (DL5: *N* = 9 flies, DM2: *N* = 9, VA1lm: *N* = 5, mean with SEM). See methods for odorant abbreviations. **(C)** We categorized responses into ***on*** (only responding during the stimulus), ***off*** (only responding after the offset of the stimulus), or ***prolonged*** (sustained responses starting with the stimulus but outlasting it), based on response thresholds (see methods for details) during the marked time points (colored squares above the graph). Dashed gray line indicates the threshold (exemplary). Black line indicates the odorant stimulus. **(D)** Fraction of glomeruli in each animal responding with ***on, off***, or ***prolonged*** time courses (*N* = 9 flies for ORN axons, *N* = 10 for PN dendrites; data pooled across animals, distribution across 6 odorants. Boxplots show median and quartiles, whiskers delimit 1.5 × interquartile range.) See Materials and Methods for number of flies and glomeruli.

**FIGURE 3:**
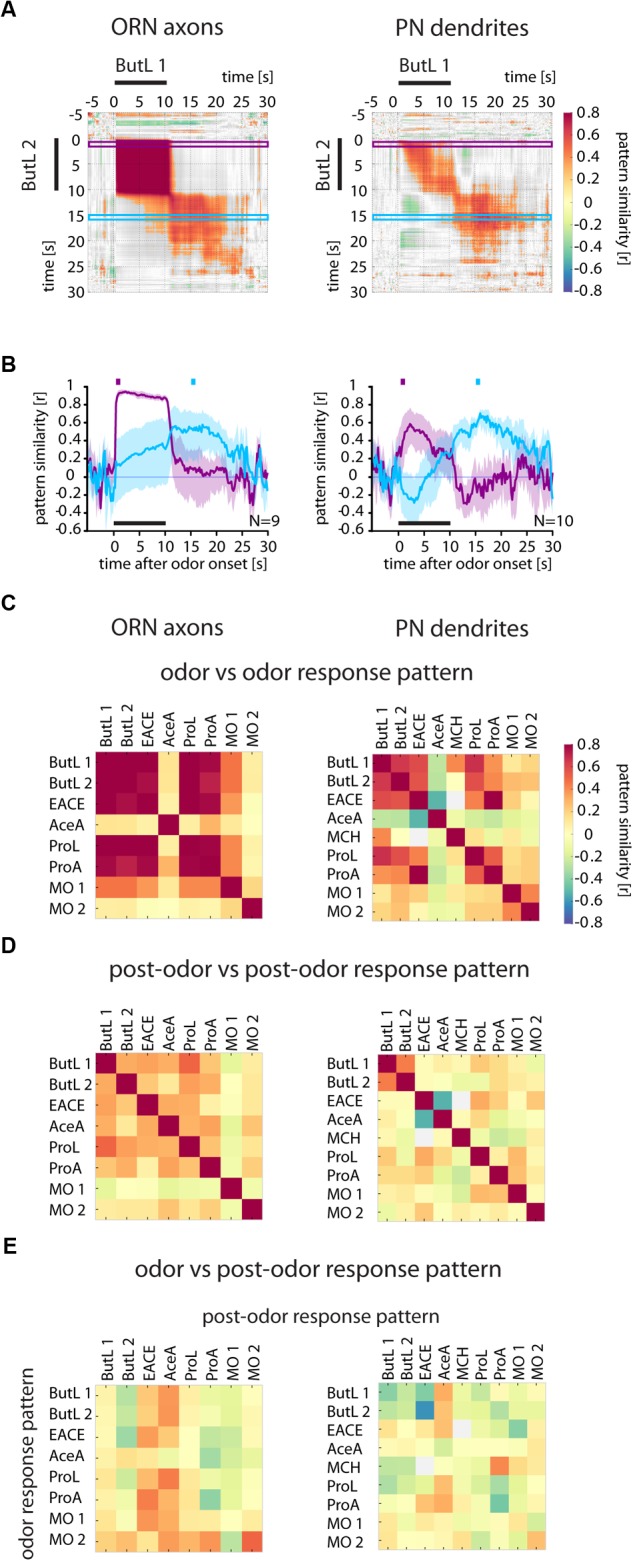
Odor response patterns in ORN axons and PN dendrites change after odor offset. **(A)** Correlation analyses of two 1-butanol (ButL) responses show that both odor and post-odor response patterns were reproducible and stable (high correlation values within the block during the odorant stimulation and within the block after odor offset), but dissimilar to each other (low correlation values when comparing odor response period to post-odor response period). Correlation values were defined as being significant when *p* < 0.005 and are color coded (color scale bar, right). Non-significant values are shown in gray. Purple and blue frames mark the time window used to calculate the time-resolved correlation, shown in (B). **(B)** Time-resolved correlation between the odor response pattern (marked by the purple square above the graph) and the post-odor response pattern (marked by the blue square) of a 1-butanol response across all time points of another 1-butanol response (purple and blue traces, respectively). The odor response pattern breaks down at odor offset in both, ORN axons (left) and PN dendrites (right) and is dissimilar to the post-odor response pattern, which evolves at odor offset. Mean ± SD obtained by bootstrap analysis (on animals, 1000 times). **(C)** Correlation of the odor response patterns between different odors. Odorant responses are reproducible (compare ButL 1 with ButL 2). In ORN axons, different odorants evoke more similar odor response patterns (e.g., EACE vs. ButL 1 and ButL 2) than in PN dendrites (right, with fewer high values at off-diagonal locations). See methods for odorant abbreviations. **(D)** Correlation of the post-odor response patterns between different odorants. Post-odor response patterns are less correlated between different odorants than odor response patterns. Post-odor responses are more reproducible in PN dendrites than in ORNs (compare ButL 1 with ButL 2). **(E)** Correlation of odor response patterns with post-odor response patterns for each odor. The odor response patterns are not correlated with the post-odor response patterns (there is no increase in correlation along the diagonal) See Materials and Methods for number of flies and glomeruli.

**FIGURE 4:**
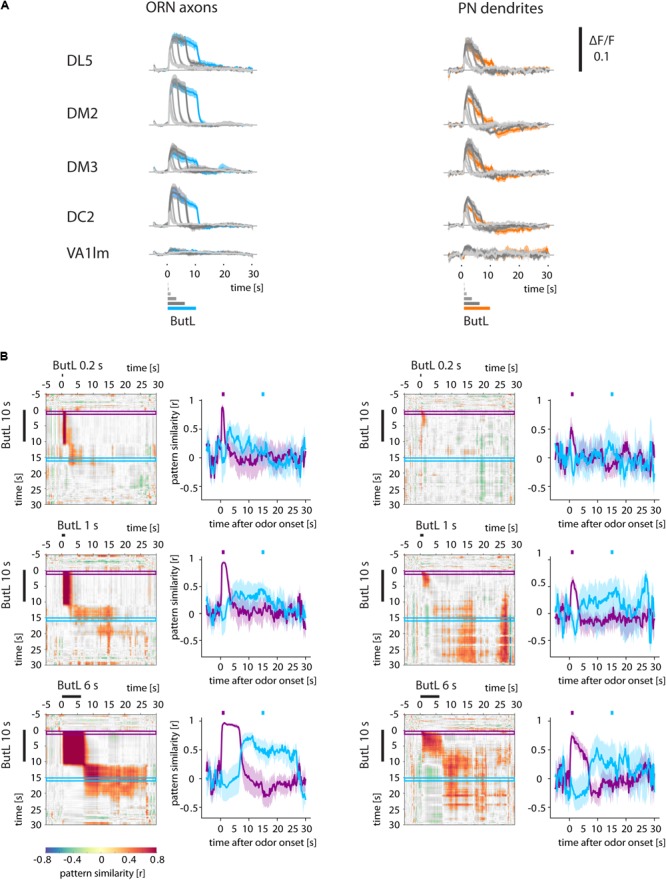
Odor and post-odor responses track stimulus length. **(A)** Ca^2+^ responses (ΔF/F) of glomeruli (ORN axons and PN dendrites) to different stimulus durations (0.2, 0.4, 1, 3, 6 s) of 1-butanol (ButL). Long stimuli lead to longer odor responses. Additionally, long stimuli also lead to more pronounced post-odor responses. **(B)** Correlation analyses comparing the glomerular response to a 10 s stimulus (vertical) with that to a shorter stimulus (horizontal, here: 0.2, 1, 6 s, for other durations see **Supplementary Figure S2**). Correlation values were defined as being significant when *p* < 0.005 and were color coded (color scale bar, right). Non-significant values are shown in gray. The correlation traces (purple and blue traces, mean ± SEM) reveal that the odor response patterns breaks down at odor offset in all measured stimulus durations and are dissimilar to the post-odor response patterns. The post-odor response patterns also increase their durations with stimulus length. See Materials and Methods for number of flies and glomeruli.

**FIGURE 5:**
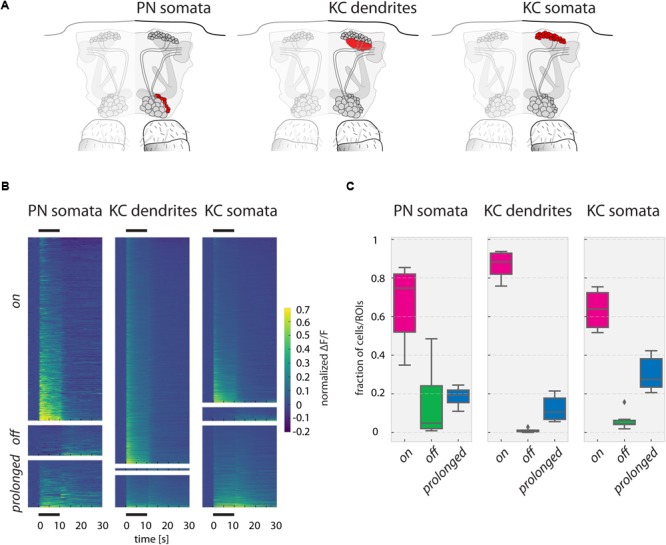
Responses to odorants in PN somata, KC dendrites, and KC somata. **(A)** Schematic of the recorded PN somata, KC dendrites in the calyx and KC somata layer in the fly brain. **(B)** Ca^2+^ responses (ΔF/F, normalized to the strongest odor response in each fly) of PN somata, KC dendrites, and KC somata of all measured odorants in all measured flies, sorted into ***on, off***, and ***prolonged*** responses. Black bars above and below the graph mark the 10 s odor stimulus. **(C)** Fraction of responding units (somata or ROIs) per odorant in each response category for all three recorded areas. ***Prolonged*** responses are significantly more frequent in KC somata than in KC dendrites (*p*-value: 0.033). Boxes show the quartiles of the datasets, whiskers extend to show the rest of the distribution, outliers are marked as ticks. See Materials and Methods for number of flies and somata/ROIs.

**FIGURE 6:**
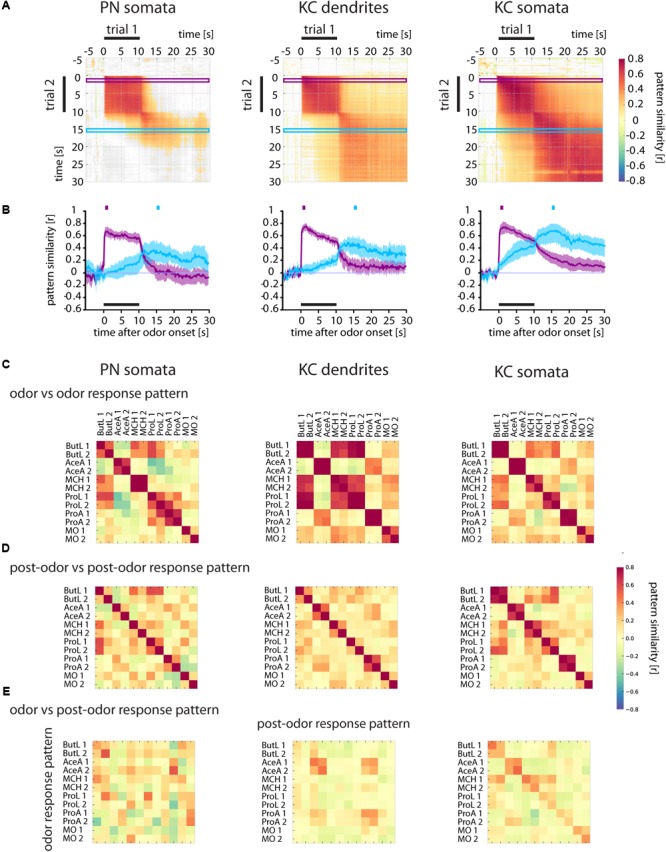
Odor and post-odor responses evolve differently in different brain areas. **(A)** Correlation analyses of repeated odorant stimulations (trial 1 vs. trial 2) show that odor and post-odor response patterns are reproducible and stable in PN somata, KC dendrites, and KC somata (see high correlation values during the odor and after odor offset). In PN somata, the odor response pattern changes into a distinct post-odor response at odor offset (see the gray areas right and below the correlated odor pattern rectangles). In KC dendrites, and even more in KC somata, the pattern change is smooth and the post-odor response pattern retains a similarity to the odor response pattern. Correlation values were defined as being significant when *p* < 0.005 and are color coded (color scale bar, right). Non-significant values are shown in gray. Purple and blue frames mark the time windows used to calculate the time-resolved correlation, shown in (B). **(B)** Time-resolved correlation between the odor response pattern (marked by the purple square above the graph) and the post-odor response pattern (marked by the blue square) of an odor response with all time points of another response to the same odorant (purple and blue traces, respectively). The odor response pattern breaks down at odor offset in PN somata, and yields to a distinct post-odor response pattern which evolves at odor offset. Conversely, KC somata show a smooth transition that retains substantial information about the odorant response also during post-odor activity. Mean ± SD obtained by bootstrap analysis (on animals, 1000 times). See **Supplementary Figure S3** for the same data split by odorants. **(C)** Correlation of the odor response patterns of different odorants and repeated odorant stimulations. Each odorant was given twice (e.g., ButL 1 and ButL 2; see methods for odorant abbreviations). Odor response patterns of repeated odorants are reproducible in all three cell/compartment types (large diagonal squares in the upper row). Some odorants elicited similar response patterns in KC dendrites (off-diagonal dark squares, e.g., ButL 1 and ButL 2 with ProL 1 and ProL 2). **(D)** Correlation of the post-odor response patterns between different odorants. Post-odor patterns are not reproducible in PN somata (high values along the diagonal form only small squares), but quite reproducible in KC dendrites and KC somata (large squares in the diagonal with high correlation values). **(E)** Correlation of odor response patterns with post-odor response patterns. In PN somata or KC dendrites, the odor response patterns are not correlated to the post-odor response patterns within the same odorant stimulation (see diagonal entries), but there is a consistent correlation in KC somata (increase in correlation along the diagonal). See Materials and Methods for number of flies and somata/ROIs.

For calcium imaging, the genetically encoded calcium sensors GCaMP1.3 (Nakai et al., 2001) or GCaMP6f (Chen et al., 2013) [Bloomington #42747, genotype w(1118); P∖y(+t7.7) w(+mC) = 20XUAS-IVS-GCaMP6f∖attP40] were expressed in ORNs using the driver line Orco-Gal4 (Larsson et al., 2004), in PNs using GH146-Gal4 (Stocker et al., 1997; Jefferis et al., 2001; Tanaka et al., 2012) and in KCs using OK107-Gal4 (Connolly et al., 1996). ORN and PN glomerular recordings (**Figures 1–4**) were performed with GCaMP1.3, PN somata and KC recordings (somata and dendrites, **Figures 1** and **5, 6**) were performed with GCaMP6f.

### *In Vivo* Calcium Imaging

Female flies (aged 2–14 days after eclosion) were prepared either for antennal lobe recordings as described previously (Silbering and Galizia, 2007; Galili et al., 2011), or for accessing the layer of KC somata from the posterior side of the fly’s head, similar to the methods described elsewhere (Murthy and Turner, 2010; Honegger et al., 2011; Campbell et al., 2013). In both preparation methods, the fly head was fixed with a composition of low melting wax (hard wax, soft-sticky wax, and myristic acid, composition ratio 2:1:2, melted and mixed), and the antennae were shielded from the Ringers’ solution with a plastic coverslip which had a window to gain access to the head capsule. Gaps between the cover slip window and the head were sealed with silicone elastomer (Kwik Sil, World Precision Instruments, Sarasota, FL, United States). Through the window the cuticle was cut, opened, and removed, so that the brain was visible.

Calcium imaging was done with experimentally naïve, unconditioned flies. Calcium imaging of ORNs and PNs in the antennal lobe was performed as described previously (Galili et al., 2011) with a fluorescence microscope (either BX-50 WI, Olympus, Tokyo, Japan, or AXIO Examiner.D1, Zeiss, Jena, Germany), equipped with a 40× water-dip objective (NA 1.0; Zeiss, Jena, Germany). The excitation wavelength (475-nm, Monochromator Polychrome II or Polychrome V, Till Photonics, Gräfelfing, Germany) was filtered with a 500-nm short-pass filter and reflected onto the sample by a 495-nm dichroic mirror. The emission light was filtered by a 505-nm long-pass filter, before being captured by the CCD-Camera (either Imago QE at the Olympus Microscope or PCO. Imaging SensiCam at the Zeiss Microscope, both: Till Photonics, Gräfelfing, Germany).

On-chip binning of pixels (4 × 4 in the Imago QE and 8 × 8 in the SensiCam) resulted in a resolution of 160 × 120 pixels, corresponding to 145 × 109 μm on the preparation (Olympus Microscope) or 172 × 130 pixels, corresponding to 242 × 183 μm on the preparation (Zeiss Microscope). The recording rate was 5 Hz for 35 s (175 frames). The exposure time was adjusted between 120 and 180 ms and the basal fluorescence was adjusted either by adding gray filters into the excitation light beam (5, 10, and 32% transmission, at the Polychrome II, Olympus Microscope), or by tuning the intensity and bandwidth of the Polychrome V monochromator (at the Zeiss Microscope). During the recording, odorant stimulation was controlled by the acquisition software of the imaging system (Till Vision, Till Photonics). Odorants were applied for 10 s in a pseudo-randomized order. The interval between recordings was 2 min (interstimulus interval, ISI, 2 min).

The KC and PN somata recordings were performed with the Zeiss LSM 510 Confocal Microscope to reduce scattered light. Excitation light was 488-nm (Argon laser), the objective 40× water-dip (NA 1.0; Zeiss, Jena, Germany). Odorant stimulation was controlled by a stimulus control device (cRIO-9074 combined with IO module NI-9403, National Instruments) with custom-written software (by Stefanie Neupert), which was synchronized by the image acquisition software. Odorants were applied for 10 s in a pseudo-randomized order. The interval between recordings (ISI) was 2 min.

### Odorants

Odorants (Sigma-Aldrich, Deisenhofen, Germany) were diluted in 10 mL of mineral oil (MO, Sigma-Aldrich) in 100 mL rolled-flange glass bottles (Fisher Scientific GmbH, Schwerte, Germany), which were sealed with silicon-Teflon septa (Schmidlin Labor Service GmbH, Schwäbisch Gmünd, Germany). The bottles were connected to a custom-built, computer-controlled olfactory stimulator (Szyszka et al., 2011) via syringes (1.2 mm external diameter) through the septum. Fresh odorant solutions were prepared every 1–4 weeks. A constant airstream (3 L min^-1^) was applied to the fly’s antennae through a glass tube (inner diameter 6.2 mm), which was located approximately 8 mm away from the fly. This constant airstream was the sum of a carrier airstream (1.2 L min^-1^) and six channels (each 0.3 L min^-1^). The olfactory stimulator produced nearly rectangular odor pulses with steep odor on- and off-sets, as measured using a photoionization detector (PID, Model 200a, Aurora Scientific Inc., **Supplementary Figure S1**). Continuous air suction behind the fly cleared residual odorants. For odorant stimulation, we used the odorants in the following dilutions: 1-butanol (CAS: 71-36-3; 1:500, ButL), 1-propanol (CAS: 71-23-8; 1:500, ProL), acetic acid (CAS: 64-19-7; 1:200, AceA), and propanoic acid (CAS: 79-09-4; 1:200, ProA) for all recordings (ORN axons, PN dendrites, PN somata, KC dendrites, and KC somata), 4-methylcyclohexanol (rac) (CAS: 589-91-3; 1:1000, MCH) for all, except ORN axon recordings, and ethyl acetate (CAS: 141-78-6; 1:1000, EACE) only for ORN axon and three PN dendrite recordings.

### Data Analysis

The imaging data were analyzed with custom routines written in Python 2.7^1^, R^2^, and IDL (RSI, Boulder, CO, United States).

### Data Preprocessing

First, the image sequences obtained by Ca^2+^ imaging were movement corrected (anatomical landmark-based for ORN and PN recordings in the antennal lobe; affine and nonlinear registration for PN and KC somata and KC dendrites) within and between measurements. The relative fluorescent change ΔF/F was calculated for each time point *i* of the recording as ΔF/F = (F_i_-F_B_)/F_B_, where F_i_ is the absolute fluorescence of the *i*^th^ frame and F_B_ is the background fluorescence, which was calculated as the average fluorescence of 15 frames before odorant stimulation (frames 10–25). Response traces were corrected for bleaching, by fitting and subsequently subtracting an exponential decay function *F*(*t*) = *ae*^bt^+*c* to the average light intensity change in each region of interest (ROI) over time (Galizia and Vetter, 2004).

### Region of Interest Selection in Imaging Data

**FIGURE 7:**
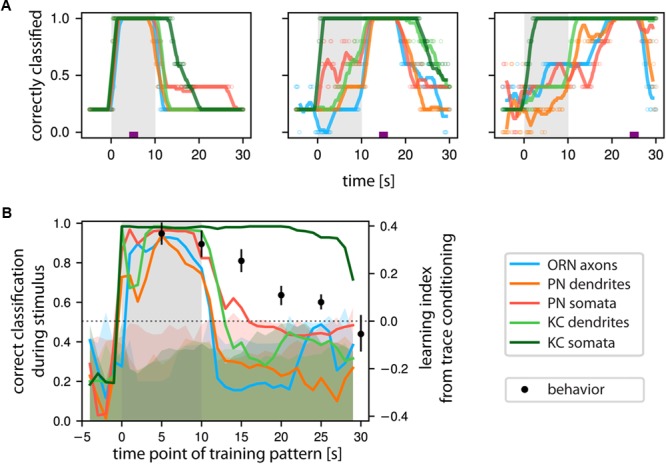
KCs’ somatic Ca^2+^ encodes odorant identity of past stimuli. **(A)** Classification success of a support vector machine (SVM) trained to correctly identify odorant identity based on the Ca^2+^ response patterns at a time point marked by the purple tick. Classification success was then evaluated at all time points. When the time points of test and train coincide, the classification performance trivially reaches 1. (Left): odor response patterns are stable over the entire stimulus time and decay with odor offset. (Middle): Training on post-odorant responses right after stimulus offset allows classifying odorant identity in KC somata, to a lesser extent in PN somata but not in the other populations. (Right): SVM trained on post-odor response patterns from KC somata successfully classified correct stimulus identity of previous responses, but not so in the other measured neuronal compartments. Individual data points are shown by empty circles, lines show a running average. Shaded area marks the odor stimulus. **(B)** Quantification of the classification success during the stimulus based on the time point of training. SVM was trained with all time points (*X*-axis), and the average correct classification during the stimulus was calculated (*Y*-axis). Shaded bars denote chance classification rates obtained by performing the analysis 250 times on label permuted datasets. The upper bound of the shaded area denotes the 95% confidence interval of such chance classification. Only KC somata were above this chance classification rate for an extended period of time. We overlaid the behaviorally observed learning performance (Data from Galili et al., 2011, with 0 learning scores coinciding to the chance level of classification, right ordinate axis). See Materials and Methods for number of flies and glomeruli/somata/ROIs.

The ROIs were determined manually in each fly using the interactive calcium signal data analysis suite ILTIS^3^ or IDL routines. Glomerular responses were calculated by averaging the fluorescent light intensity of 7 × 7 pixels (corresponding to 6.3 × 6.3 μm) in the center of a glomerulus. Glomeruli were identified based on anatomical cues and on their response profiles (Silbering and Galizia, 2007; Silbering et al., 2008; Galili et al., 2011). For single soma resolution imaging, somata responding to at least one of the five odorants during the whole recording were selected. The choice of whether a soma was active or not was based on visual observation, but conservative in the sense that even very weakly responding cells were selected. This means that somata that did not respond to any of the odorants could not be selected, resulting in an overestimate of responding cells as compared to when all KCs are considered. ROI size was 8–10 pixel diameter and placed centrally on the soma. KC dendrites in the calyx did not show any clear pattern of single cell resolution. Hence, we spatially downsampled the recordings and treated each 4 × 4 pixel bin as one ROI. Only ROIs with a signal larger than 0.75 ΔF/F to any of the 5 odorants were included in later analyses. The following number of flies and glomeruli/somata/ROIs were used in the analyses:

**Figures 2, 3, 7**:
  ORN axons: *N* = 9 flies, *n* = 85 glomeruli (glomeruli per fly: 11, 11, 10, 5, 10, 10, 7, 10, 11).
  PN dendrites: *N* = 10 flies, *n* = 88 glomeruli (glomeruli per fly: 9, 5, 8, 9, 11, 10, 10, 12, 7, 7).
  (In **Figures 3C–F** the *N* and *n* for the odors EACE (*N* = 3, *n* = 22) and MCH (*N* = 7, *n* = 66) in PN dendrites is lower, since these odors were used alternately.)
**Figure 4 and Supplementary Figure S2**:
  (same flies as above, with one additional fly and thirteen additional glomeruli in ORN axons):
  ORN axons: *N* = 10 flies, *n* = 98 glomeruli (glom. per fly: 11, 11, 10, 6, 10, 10, 10, 9, 10, 11).
  PN dendrites: *N* = 10 flies, *n* = 88 glomeruli (glom. per fly: 9, 5, 8, 9, 11, 10, 10, 12, 7, 7).
**Figures 5, 6**:
  PN somata: *N* = 10 flies, *n* = 108 somata (somata per fly: 18, 15, 13, 5, 13, 10, 9, 9, 12, 4).
  KC dendrites: *N* = 6 flies, *n* = 343 ROIs (ROIs per fly: 57, 35, 31, 60, 84, 76).
  KC somata: *N* = 9 flies, *n* = 339 somata (somata per fly: 47, 28, 26, 52, 44, 23, 3, 55, 61).
  (In **Figures 6C–F** and **Supplementary Figure S3** the *N* and *n* in the PN somata and KC somata matrices vary for each odor pair, because not every odor was analyzable in every fly. PN somata: *N* = 4–10 flies, *n* = 47–108 somata; KC somata: *N* = 5–8 flies, *n* = 217–313 somata.)
**Figure 7**:
  PN somata: *N* = 2 flies, *n* = 25 somata (somata per fly: 13, 12).
  KC dendrites: *N* = 6 flies, *n* = 343 ROIs (ROIs per fly: 57, 35, 31, 60, 84, 76).
  KC somata: *N* = 5 flies, *n* = 217 somata (somata per fly: 47, 28, 26, 55, 61).
  (Note that for the SVM we could only use flies with complete data for the same set of odorants (ButL, AceA, ProL, ProA, MO), hence the lower N in PN somata and KC somata.)

### Categorization of Response Dynamics

In order to categorize calcium responses according to their response dynamics, we formed three categories into which a response could fall: ***on*** (only responding during the stimulus), ***off*** (only responding after the offset of the stimulus), or ***prolonged*** (sustained responses starting with the stimulus but outlasting it) (**Figure 2C**). For our categorization, we defined thresholds that allowed us to unambiguously classify each recorded response into one of the three categories: We defined ***on*** or ***prolonged*** responses those which crossed a threshold of 2.57 × SD of the pre-stimulus signal (corresponding to a significance level of *p* < 0.005). If they declined below 37% (corresponding to 1/e) of the maximum response value within 5 s after odorant offset, they were classified as ***on***, otherwise as ***prolonged***. We defined ***off*** responses those which crossed the response threshold 5 s after odorant offset but not during the stimulus.

### Correlation Matrices and Traces

Time-resolved correlation matrices were constructed to compare the change of patterns and their similarity during the recording. This was done by first combining data from all animals for either a given odorant (as in **Figure 3** for 1-butanol and **Supplementary Figure S3**) or all odorants (as in **Figure 6**) into two matrices, separately for the two individual stimulations. The Pearson’s correlation coefficient *r* between each pair of response pattern vectors for all time points was then calculated. The color scale represents correlation values with *p* < 0.005, while correlation values with *p* > 0.005 are displayed in gray. Correlation traces (**Figures 3B, 4B, 6B** and **Supplementary Figure S2**) were extracted from the correlation matrices by plotting the average correlation during 1 s of the odor response or post-odor response over the entire recording time. They contain additional information about variability across stimulations: To obtain this estimate of the variability in our correlation analysis, we performed a bootstrapping analysis by resampling the animals included in our analysis 1000 times. Shaded areas show the SD of the bootstrapped values.

### Classification Analysis

Classification analysis was performed by training a support vector machine (SVM) with a 2-s averaged response pattern sliding across the total time of the recording, and testing all other time points afterwards. To assess overall classification performance, the average fraction of correct classifications during the 10 s stimulus window was calculated. All SVM classifications were performed with a ν-SVM (python module sklearn.svm.NuSVC with a ν-parameter of 0.9). Chance classification levels were obtained by shuffling all labels and repeating the classification 250 times, and the upper 95% confidence interval is taken as a threshold for significant classification (Combrisson and Jerbi, 2015).

## Results

We studied the odor-specificity of odorant-evoked activity during and after olfactory stimulation in different neuronal populations and compartments in the fly brain. We measured odorant-evoked Ca^2+^ concentration changes in ORN axons, PN dendrites, and PN somata (in the antennal lobes) and in KC dendrites and KC somata (in the mushroom bodies) (**Figure 1A**). Stimulus onset was defined as *t* = 0 s. Responses during stimulation were called “odor response,” activity after stimulation “post-odor response” (**Figure 1B**). We defined “odor response pattern” as the averaged response of 5 frames from *t* = 1–2 s, and “post-odor response pattern” as the 5 frames from *t* = 15–16 s (**Figure 1B**, bottom). We chose this time point as post-odor response pattern because trace conditioning experiments have shown that flies still have a sensory memory of the odorant at this time point (Galili et al., 2011). We categorized Ca^2+^ responses according to their response dynamics, forming three threshold-based categories: ***on*** (only responding during the stimulus), ***off*** (only responding after the offset of the stimulus), or ***prolonged*** (sustained responses starting with the stimulus but outlasting it) (**Figure 2C**, see methods for details on the construction of thresholds used for categorization).

### Glomerular Post-odor Response Patterns in the Antennal Lobe Are Dissimilar to Odor Response Patterns

The Ca^2+^ responses in ORN axons and PN dendrites (**Figure 2A**) in the antennal lobe were odor- and glomerulus-specific (**Figure 2B**), with a tendency of PN responses to be more phasic than ORN responses and with more pronounced off-responses. Across glomeruli, axonal ORN activity patterns and dendritic PN response patterns generally involved the same glomeruli, in line with previous publications (Wang et al., 2003; Silbering and Galizia, 2007). In most glomeruli (three example glomeruli are shown in **Figure 2B**), positive responses decreased rapidly after odorant offset and reached baseline within our recording time of 20 s for both ORNs and PNs. Acetic acid (AceA) and propanoic acid (ProA) induced negative responses in some glomeruli during the stimulus, which were sometimes followed by positive off responses.

The diversity of response dynamics across odorants was large in ORNs, with all three categories (***on, off***, and ***prolonged***) taking up a large share (medians of 25, 44, and 27% for ***on, off***, and ***prolonged***, respectively, **Figures 2C,D**). Conversely, ***on*** responses dominated in PNs (75%, **Figure 2D**).

As the spatial activity pattern across glomeruli contains information about odorant identity (Guerrieri et al., 2005), a stable activity pattern over an extended period of time after stimulus offset would be indicative of a possible sensory memory. Thus, we performed a spatiotemporal analysis by calculating the correlation between stimulus evoked activity patterns along the entire recording (**Figure 3A**). The resulting time-resolved correlation matrices (**Figure 3A**) show the correlation between the response patterns at each single time point of each recording, where each pixel displays the Pearson’s correlation coefficient *r* of the two response vectors at these particular time points.

For ORNs, the odorant-evoked glomerular patterns during the 10-s-long stimuli were correlated (as shown by the compact dark red “field” in the plot of **Figure 3A**), showing that the odorant-evoked patterns were largely invariant during the entire stimulation period and reproducible in different trials. However, the activity patterns after stimulus offset were not correlated to the odor responses anymore (**Figure 3A**, gray areas to the right and below the odor response “field”).

Although post-odor response patterns in ORNs were not correlated to the odor response patterns, they were fairly stable within themselves (**Figure 3A**). However, they were neither odorant-specific nor reproducible (no increased correlation between the post-odor response patterns of two 1-butanol trials in **Figure 3D**).

Next, we analyzed how the spatiotemporal odor response pattern (*t* = 1–2 s) and the post-odor response pattern (*t* = 15–16 s, i.e. 5 s after odorant offset) developed over time. To this end, we calculated the correlation to these time windows across different trials (**Figure 3B**, purple and blue frames in **Figure 3A** mark the correlation values plotted in 3B). We found that the ORN odor response pattern (purple trace) was stable during the odor stimulus, but immediately collapsed at odorant offset. On the other hand, the post-odor response pattern (blue trace) gradually evolved into a distinct and dissimilar pattern.

Similarly, PN dendrite response patterns were also correlated between two 1-butanol stimulations, but when compared to ORNs the correlation values were lower and decreased during the 10-s stimulation (**Figures 3A,B**). This shows that the odor response pattern of PN dendrites was both less stable during odorant stimulation and less reproducible from trial to trial. After odorant offset, the post-odor response pattern was not correlated to the odor response pattern (**Figures 3A,B**).

Across different odorants, we found that similarity was higher in ORNs compared to PNs (**Figures 3C–E**). This confirms previous observations of higher odorant-specificity in PNs (Olsen and Wilson, 2008; Silbering et al., 2008) as well as higher response variability in PNs (Jeanne and Wilson, 2015). However, for all tested odors and both ORNs and PNs, the correlations between the odor and post-odor response patterns were low (diagonal entries, **Figure 3E**). This indicates that no information about odor identity was maintained in the glomerular response pattern after odorant offset, and ORNs and PNs do not contain any sensory memory in their Ca^2+^ response activity.

Do post-odor responses of ORN axons and PN dendrites in the antennal lobe depend on the stimulus duration? We compared post-odor responses after a 10-s-long stimulus with post-odor responses after shorter stimuli (0.2, 0.4, 1, 3, and 6 s) (**Figure 4** and **Supplementary Figure S2**). In both ORN axons and PN dendrites, stimuli of different length activated the same glomeruli (**Figure 4A**), and accordingly odor response patterns were correlated (**Figure 4B** and **Supplementary Figure S2**). After odorant offset, Ca^2+^ responses of both ORNs and PNs rapidly changed for all stimulus durations. Additionally, post-odor response patterns for different stimulus durations were more similar to each other with increasing stimulus durations (**Figure 4B** and **Supplementary Figure S2**).

### Post-odor Response Patterns in KCs Are Similar to Odor Response Patterns

The Ca^2+^ responses in PN dendrites consist of both pre- and post-synaptic activity (Ca^2+^influx through cholinergic receptors and Ca^2+^ influx through voltage-gated Ca^2+^channels). In the cell bodies (somata), however, Ca^2+^ influx is driven by cell depolarization only, and thus reflects a further processing step with respect to PN dendrites. However, PN somata cannot be attributed to identified glomeruli, and therefore, we analyzed patterns across non-identified “anonymous” units. Similarly, we analyzed “anonymous” responses in the mushroom bodies, recording KC dendrites and KC somata (**Figures 5A,B**). As before, we categorized the responding cells/ROIs again into ***on, off***, and ***prolonged*** responding units (**Figures 5B,C**). In all three cell/compartment types, most units showed ***on*** responses. ***Off*** responses were rare in PN and KC somata, and almost nonexistent in the KC dendritic region. KC somata had a significantly larger proportion of ***prolonged*** responses than KC dendrites. Many of the observed ***prolonged*** responses, particularly in PN and KC somata, lasted even longer than the 20 s recording time after odorant offset (**Figure 5B**).

To investigate whether the ***prolonged*** responses in PN somata, KC dendrites and KC somata maintained the odor-specific pattern after odorant offset, we performed the same correlation analyses as done for glomerular signals and quantified the time-resolved pattern similarity between two stimulations (trial 1 and trial 2) of the same odorant (**Figure 6A** and **Supplementary Figure S3**).

Similar to the 1-butanol responses in PN dendrites (**Figure 3A**), odor response patterns and post-odor response patterns in their somata (PN somata) were reproducible and odor-specific (**Figure 6A** and **Supplementary Figure S3**). However, odor response patterns were uncorrelated to post-odor response patterns (**Figure 6A** and **Supplementary Figure S3**). Odor response patterns (purple trace, **Figure 6B**) broke down within 3–4 s after odorant offset, while the post-odor response pattern (blue trace) developed after the end of the odorant stimulation and lasted for several seconds. This suggests that it is rather unlikely that Ca^2+^ activity in PN somata harbors a sensory memory for specific odors.

In KC dendrites, the odor response patterns and post-odor response patterns were reproducible over stimulus repetitions (**Figure 6A**). Unlike all upstream compartments, the post-odor response patterns were correlated to the odor response pattern (**Figure 6A**). The correlation trace (**Figure 6B**, purple trace) showed a decline of pattern similarity within 3 s after odorant offset (correlation values from 0.5 to < 0.2), but did not reach baseline. This indicated that Ca^2+^ activity in KC dendrites maintained a pattern correlation to the odor response pattern after odorant offset, which could contribute to a sensory memory.

Similar to the other analyzed cellular compartments, KC somata also showed reproducible odor and post-odor response patterns (**Figure 6A** and **Supplementary Figure S3**). Here, we found an elevated similarity between post-odor and odor response patterns: After odorant offset, the similarity to the odor response patterns changed only gradually, maintaining a correlation to the odor response pattern (**Figures 6A,B**, purple trace). Furthermore, the post-odor response pattern already developed during the odorant stimulation (**Figure 6B**, blue trace). This pattern remained correlated to the odor response pattern for at least 20 s after odorant offset (until the end of the recording time), i.e., for a behaviorally relevant time scale (Galili et al., 2011). Together, these results indicated that Ca^2+^ activity in KC dendrites and even more in KC somata had an elevated similarity between odor and post-odor response pattern. This Ca^2+^ activity could represent the neural substrate for a sensory memory in trace conditioning.

### Post-odor Response Patterns Are More Reproducible and Odorant-Specific in KC Somata

How specific were the Ca^2+^ responses for each odor? We analyzed the correlation matrices for all odor pairs for the odor response patterns across trials (*t* = 1–2 s, **Figure 6C**) and the post-odor response patterns across trials (*t* = 15–16 s, **Figure 6D**) (for the corresponding correlation matrices over time of the single odor repetitions see **Supplementary Figure S3**). The odor response patterns were reproducible across trials for all tested odorants (**Figure 6C** and **Supplementary Figure S3**), as shown by the high values for repeated odorant stimulations in all three compartments. Compared to KC somata, the correlation between odor response patterns for repeated stimulus presentations was lower in PN somata (except 4-methylcyclohexanol, MCH). Post-odor response patterns increased in their reproducibility from PN somata, over KC dendrites to KC somata (**Figure 6D**), indicating that the neural networks in the brain modify response patterns towards more stereotypic, odor-specific activity patterns.

If post-odor responses encode an odorant-specific sensory memory, then those post-odor response patterns should be similar to the immediate odor-responses. We therefore quantified the pattern similarity between odor responses and post-odor responses (**Figure 6E**). There was no such correlation for PN somata. For KC dendrites, correlations were elevated for acetic acid (AceA) and propanoic acid (ProA) (both within and between odorants and stimulus repetitions). In KC somata, however, the correlations were odorant-specific and reproducible for most odorants (**Figure 6E**). Thus, KC somata post-odor response patterns could encode an odorant-specific sensory memory. Their post-odor responses were most reproducible, most odorant-specific and most similar to the corresponding odor response patterns as compared to all other compartments studied here.

### Prolonged Response Patterns in KC Somata Can Identify the Past Odors’ Identity

In olfactory trace conditioning, insects learn the association between the odorant and the reinforcer during the post-odor response (because this is when the reinforcer is given), but during test they respond to the actual odorant stimulus (Galili et al., 2011; Szyszka et al., 2011). Therefore, when the brain forms an associative memory between an already terminated odorant stimulus and a current punishment or reward, the post-odor activity pattern must allow the recognition of future presentations of the same odorant. Therefore, we asked whether the activity patterns in the measured cell compartments contained sufficient information to identify the odor response pattern itself. We trained a SVM with the response pattern during odor presentation, 5 s after odor offset, or 15 s after odor offset, and then tested for correct stimulus identification over time (**Figure 7A**). When trained with the odor response patterns, classification was strong for all compartments during the stimulus and decayed shortly after (**Figure 7A**, left panel). When trained with a post-odor response pattern, most neuronal compartments’ patterns were not suitable to recognize the odor response pattern (**Figure 7A**, middle panel). However, the SVM trained on the KC somata post-odor responses resulted in high classification also during the odor responses (**Figure 7A**, middle and right panel).

Training the SVM with a sliding window and testing with the odor response pattern confirmed these results: after stimulus offset, odorant classification by ORNs, PNs, and also KC dendrites quickly dropped to or below chance level. However, odorant classification by KC somata remained high for at least 20 s (dark green line in **Figure 7B**). We compared the resulting time-course of the odorant classification success to behavioral learning performance during trace conditioning, where the reinforcer (punishment) was given at different time points after odorant onset (*X*-axis in **Figure 7B**). Learning was quantified as the choice between the persistent odorant stimulus and a solvent after conditioning (right *Y*-axis in **Figure 7B**). We found that the SVM odorant classification performance closely reproduced the behavioral odorant recognition performance (Galili et al., 2011).

## Discussion

We measured odorant-evoked cytosolic Ca^2+^ signals in multiple successive layers of the olfactory system in *Drosophila*: in ORN axons, PN dendrites, PN somata in the antennal lobes, and KC dendrites and KC somata in the mushroom body calyx. In all neurons and neuronal compartments, we recorded the Ca^2+^ responses to a common set of odorants. Our analysis focuses on the evolution of the spatiotemporal response patterns and their odorant specificities during and after the odorant stimulus.

### Transformation of Immediate Odorant Responses Along the Olfactory Pathway

We found that the stimulus-specificity and separability of the immediate odorant-evoked response patterns across neurons successively increased with each processing layer (**Figures 3C, 6C**). This is in accordance with previous reports that found lateral inhibition via GABAergic interneurons increased odorant-specificity of PNs (Olsen and Wilson, 2008; Silbering et al., 2008). The combination of a divergent projection of PNs to KCs and feedback inhibition of KCs leads to the formation of sparser and more odorant-specific KC ensemble responses, as compared to PN ensemble responses (Szyszka et al., 2005; Honegger et al., 2011; Lin et al., 2014). These findings are also in line with theoretical predictions for pattern based feature extraction systems, where such a divergence-convergence motif leads to increased pattern separability (Luo et al., 2010; Stevens, 2015). Note that the Ca^2+^ signals in ORN axons and PN dendrites in the antennal lobe and in KC dendrites in the mushroom body originate from multiple, overlapping neurons. In the antennal lobe, all ORNs or PNs that innervate the same glomerulus have highly correlated response profiles (Kazama and Wilson, 2009). Therefore, the glomerular Ca^2+^ signals in ORNs and PNs reflect the response of individual ORNs and PNs. However, the dendritic Ca^2+^ signals in KCs reflect the summed activity of different KCs with different response properties. Indeed, the dendritic KC responses were less odorant-specific than the response of individual KC somata (**Figure 6C**).

### Post-odor Responses in Kenyon Cell Somata Encode a Sensory Odor Memory

After stimulus offset, we found ongoing odorant-specific activity (**Figures 3, 6**). This has been reported for both the insect antennal lobe (Stopfer et al., 2003; Galili et al., 2011; Szyszka et al., 2011; Saha et al., 2017) and the vertebrate olfactory bulb (Patterson et al., 2013). Even though odorant-specific, this ongoing activity was different from the early response in ORNs and PNs (**Figure 3A**). This is in line with previous reports [ORNs: (Galili et al., 2011); PNs: (Nawrot, 2012; Szyszka et al., 2011; Saha et al., 2017)].

Post-odor responses also occurred in the mushroom body in the dendrites and somata of KCs (**Figure 6**). Compared to PN somata and KC dendrites, KC somata had the largest fraction of prolonged responses (**Figures 5B,C**). In contrast to PN somata, the KC somata prolonged responses were stimulus-specific (**Figure 6D**).

To analyze whether these post-stimulus responses are informative about the odorant identity, we used a classifier-based decoding analysis to predict the identity of past odorant stimuli in a time resolved manner. With this approach, we found that only the Ca^2+^ signals of the KC somata allowed predicting the previously presented odorant’s identity on a behaviorally relevant time scale (**Figure 7B**). Thus, cytosolic Ca^2+^ responses in KC somata contain all the information necessary to encode odorant identity after the stimulus offset. This shows that cytosolic Ca^2+^ forms a potential substrate for short-term sensory odor memory.

Animals need sensory odor memories in many situations. Such a memory is required for odor plume-tracking when insects continue their odor source search after losing an attractive odor plume (Vickers, 2005; van Breugel and Dickinson, 2014; Saxena et al., 2017), or for the olfactory working memory in honey bees when they solve delayed-matching-to-sample tasks (Giurfa et al., 2001), or to learn associations between temporally separated stimuli, as in trace conditioning (Galili et al., 2011; Szyszka et al., 2011). Our data show that Ca^2+^ levels in KC somata are a suitable substrate for such sensory odor memories. Our data do not exclude that Ca^2+^ signals in other neurons, not measured here, or signals other than Ca^2+^ may encode sensory odor memories (Galán et al., 2006; Dylla et al., 2013; Betkiewicz et al., 2017).

### Mechanisms of Prolonged Ca^2+^ Levels in Kenyon Cell Somata

Which cellular and molecular mechanisms could cause the prolonged Ca^2+^ elevation? A current analysis of cockroach KCs by Demmer and Kloppenburg (2009) found Ca^2+^ currents and Ca^2+^-dependent currents that are unusual for insect neurons: the same neurons had both low voltage-activated inward Ca^2+^ currents and high voltage-activated Ca^2+^-dependent repolarizing currents, both of large amplitude. These low voltage-activated, inward Ca^2+^ currents could lead to a nonlinear Ca^2+^ increase in response to odorant-induced post-synaptic potentials, and the duration of the post-odor Ca^2+^ response might reflect the time required to restore baseline Ca^2+^ concentrations by Ca^2+^ buffers and pumps. The high voltage-activated Ca^2+^-dependent repolarizing currents could mediate the typical fast adaptation of KC spike responses to odorants (Ito et al., 2008; Turner et al., 2008; Demmer and Kloppenburg, 2009; Palmer et al., 2013; Saha et al., 2013; Gupta and Stopfer, 2014; Kropf and Rössler, 2018). Indeed, in a modeling study, Betkiewicz et al. (2017) predicted that odorant-induced prolonged Ca^2+^ responses exist in KCs, and they suggested that the prolonged Ca^2+^-dependent repolarizing currents mediate adaptation and encode a sensory short-term memory for odorants in KCs.

### The Role of Ca^2+^ in Associative Learning During Classical Conditioning

Classical conditioning requires the detection of a predictive temporal relationship between a stimulus and a reinforcer (punishment or reward). In the standard *Drosophila* olfactory conditioning paradigm, the molecular component detecting the immediate odorant-reinforcer coincidence is thought to be the adenylyl cyclase *rutabaga* in KCs (Dudai et al., 1983; Levin et al., 1992): here, the odor information is encoded by the cytosolic Ca^2+^ in KCs, and the reinforcer is encoded by the activation of dopamine receptors in the KC axons (Gervasi et al., 2010). The coincident increase in Ca^2+^ and dopamine receptor activation induces a change in synaptic strength between KCs and mushroom body output neurons (McGuire, 2001; Schwaerzel et al., 2002; Tomchik and Davis, 2009; Gervasi et al., 2010; Séjourné et al., 2011; Aso et al., 2014; Cohn et al., 2015; Hige et al., 2015; Owald et al., 2015). However, this mechanism cannot explain trace conditioning.

Trace conditioning differs from the standard classical conditioning paradigm in that the odorant and reinforcer are separated by a temporal gap (Galili et al., 2011; Szyszka et al., 2011). Thus, a sensory odor memory is required to bridge the gap between odorant stimulus and reinforcer. The phasic nature of odorant-evoked KC spiking and KC Ca^2+^ activity in the lobes is unsuitable to encode a sensory memory (Ito et al., 2008; Turner et al., 2008; Saha et al., 2013; Gupta and Stopfer, 2014; Dylla et al., 2017). Standard conditioning and trace conditioning also differ in the underlying neuronal mechanisms: in vertebrates, different brain regions are involved in standard and trace conditioning (Solomon et al., 1986; Woodruff-Pak and Disterhoft, 2008), and different molecular constituents have been identified in *Drosophila*, where *rutabaga* is not necessary for trace conditioning (Shuai et al., 2011).

Could the prolonged and odorant-specific Ca^2+^ signals in the KC somata serve as a sensory odor memory in trace conditioning? Our data demonstrate that Ca^2+^ activity patterns across KC somata encode the necessary odorant information, and therefore could serve as a substrate for sensory odor memory during trace conditioning. Our experiments did not address the molecular nature of a possible coincidence detector. One possibility could be that a protein kinase C (Choi et al., 1991) or a non-rutabaga adenylyl cyclase (Adams et al., 2000) in the KC somata would act as coincidence detector for odorant-induced Ca^2+^ signaling and reinforcer-induced dopamine signaling. Dopaminergic neurons (PPL2ab neurons) innervate the mushroom body calyx (Mao and Davis, 2009), and application of dopamine increases cAMP in KCs in the calyx (Tomchik and Davis, 2009). Paired application of acetylcholine (simulating the odor stimulus) and dopamine synergistically increases cAMP in KCs in the lobes, but not in the calyx (Tomchik and Davis, 2009). However, it has not been tested whether the pairing of a prolonged Ca^2+^ signal with a delayed dopamine input (as is the case in trace conditioning) would induce a synergistic cAMP response. It is therefore conceivable that a non-rutabaga adenylyl cyclase (Adams et al., 2000) in the calyx might serve as a coincidence detector for the odorant-induced prolonged Ca^2+^ signal and for the reinforcer-induced dopamine receptor activation in trace conditioning.

## Conclusion

Our data suggest that, in addition to the molecular separation (Shuai et al., 2011), trace and standard conditioning in *Drosophila* might be spatially separated, reminiscent of the situation in vertebrates (Solomon et al., 1986; Woodruff-Pak and Disterhoft, 2008). This adds an interesting thought to how memories are organized in brains: seemingly equivalent memories (here, the association of an odorant with a reinforcer) might be localized in parallel and different compartments, reflecting the mode with which they were learned (trace or standard conditioning).

The molecular and spatial separation of trace and standard conditioning opens the possibility to further dissect the physiological and molecular processes underlying trace conditioning, including specific phenotypes such as *Rac* (Shuai et al., 2011), using pharmacological alterations during *in vivo* imaging, or optogenetical shunting of KC activity at different time points during a trace-conditioning paradigm. Such studies, readily performed in *Drosophila*, will lead to a more mechanistic understanding of a brain’s capability to bridge temporal gaps between stimuli and form associations across them.

## Author Contributions

AL performed the experiments. AL, PS, and CG designed the experiments. GR and AL analyzed the data. JN and AH advised about the data analysis. AL, GR, PS, and CG wrote the paper. All the authors edited the paper.

## Conflict of Interest Statement

The authors declare that the research was conducted in the absence of any commercial or financial relationships that could be construed as a potential conflict of interest.

## Footnotes

1 www.python.org

2 www.r-project.org

3 https://github.com/grg2rsr/ILTIS

## Supplementary Material

FIGURE S1 The olfactory stimulator produced odorant pulses, with steep odorant on- and off-sets. Traces show photoionization detector signals (PID, Model 200a, Aurora Scientific Inc.) during stimulation with the tracer substance ethyl acetate (undiluted, ionization potential 10.01 eV) for different pulse lengths (0.2, 0.4, 1, 3, 6 s in channel 1, and 10 s in channel 2; indicated by the bar below each graph, single measurements). For PID traces to other odorants applied with a similar olfactory stimulator, see (Raiser et al., 2017).

FIGURE S2 Related to **Figure 4**: Post-odor response patterns of ORN axons and PN dendrites differ from odor response patterns. Corresponding graphs as in **Figures 3A,B**, for cross correlations between stimuli of increasing length (abscissa, from top to bottom), with a stimulus of 10 s length (ordinate, in each graph).

FIGURE S3 Related to **Figure 6**: Odor and post-odor responses evolve differently in different brain areas. Corresponding graphs as in **Figure 6** for cross correlations between responses to repeated presentation of the same odorant, in PN somata, KC dendrites and KC somata [columns **(A–C)**, respectively]. See methods for odorant abbreviations.
