# Corrigendum: Calcium in Kenyon Cell Somata as a Substrate for an Olfactory Sensory Memory in *Drosophila*

**DOI:** 10.3389/fncel.2018.00197

**Published:** 2018-07-03

**Authors:** Alja Lüdke, Georg Raiser, Johannes Nehrkorn, Andreas V. M. Herz, C. Giovanni Galizia, Paul Szyszka

**Affiliations:** ^1^Department of Biology, Neurobiology, University of Konstanz, Konstanz, Germany; ^2^International Max Planck Research School for Organismal Biology, Konstanz, Germany; ^3^Fakultät für Biologie, Ludwig-Maximilians-Universität München, Martinsried, Germany; ^4^Bernstein Center for Computational Neuroscience, Munich, Germany

**Keywords:** *Drosophila melanogaster*, olfaction, sensory memory, mushroom body, Kenyon cells, trace conditioning, calcium imaging

In the original article, we did not indicate the number of analyzed animals and glomeruli/somata/ROIs. We provide this information below:

**Figures 2, 3, and 7:**
ORN axons: *N* = 9 flies, *n* = 85 glomeruli (glomeruli per fly: 11, 11, 10, 5, 10, 10, 7, 10, 11)
PN dendrites: *N* = 10 flies, *n* = 88 glomeruli (glomeruli per fly: 9, 5, 8, 9, 11, 10, 10, 12, 7, 7)
[In Figures 3C–F the N and n for the odors EACE (*N* = 3, *n* = 22) and MCH (*N* = 7, *n* = 66) in PN dendrites is lower, since these odors were used alternately].
**Figure 4 and Supplementary Figure S2:**
(same flies as above, with one additional fly and thirteen additional glomeruli in ORN axons):
ORN axons: *N* = 10 flies, *n* = 98 glomeruli (glom. per fly: 11, 11, 10, 6, 10, 10, 10, 9, 10, 11)
PN dendrites: *N* = 10 flies, *n* = 88 glomeruli (glom. per fly: 9, 5, 8, 9, 11, 10, 10, 12, 7, 7)
**Figures 5, 6:**
PN somata: *N* = 10 flies, *n* = 108 somata (somata per fly: 18, 15, 13, 5, 13, 10, 9, 9, 12, 4)
KC dendrites: *N* = 6 flies, *n* = 343 ROIs (ROIs per fly: 57, 35, 31, 60, 84, 76)
KC somata: *N* = 9 flies, *n* = 339 somata (somata per fly: 47, 28, 26, 52, 44, 23, 3, 55, 61)
(In Figures 6C–F and **Supplementary Figure S3** the N and n in the PN somata and KC somata matrices vary for each odor pair, because not every odor was analyzable in every fly. PN somata: *N* = 4–10 flies, *n* = 47–108 somata; KC somata: *N* = 5–8 flies, *n* = 217–313 somata).
**Figure 7:**
PN somata: *N* = 2 flies, *n* = 25 somata (somata per fly: 13, 12)
KC dendrites: *N* = 6 flies, *n* = 343 ROIs (ROIs per fly: 57, 35, 31, 60, 84, 76)
KC somata: *N* = 5 flies, *n* = 217 somata (somata per fly: 47, 28, 26, 55, 61)
(Note that for the SVM we could only use flies with complete data for the same set of odorants (ButL, AceA, ProL, ProA, MO),
hence the lower N in PN somata and KC somata).

In the original article the following reference was incorrectly cited as “unpublished”. The corrected reference appears below:

Betkiewicz, R. L., Lindner, B., and Nawrot, M. P. (2017). Circuit and cellular mechanisms facilitate the transformation from dense to sparse coding in the insect olfactory system. *BioRxiv* [Preprint]. doi: 10.1101/240671

We apologize for this missing information and emphasize that this does not change the scientific conclusions of the article in any way.

The original article has been updated.

## Conflict of interest statement

The authors declare that the research was conducted in the absence of any commercial or financial relationships that could be construed as a potential conflict of interest.

