# Supplementary figures and images for: Calcium in Kenyon Cell Somata as a Substrate for an Olfactory Sensory Memory in Drosophila
Source: Front Cell Neurosci. 2018 May 14;12:128. doi: 10.3389/fncel.2018.00128 (PMC5960692; doi:10.3389/fncel.2018.00128)

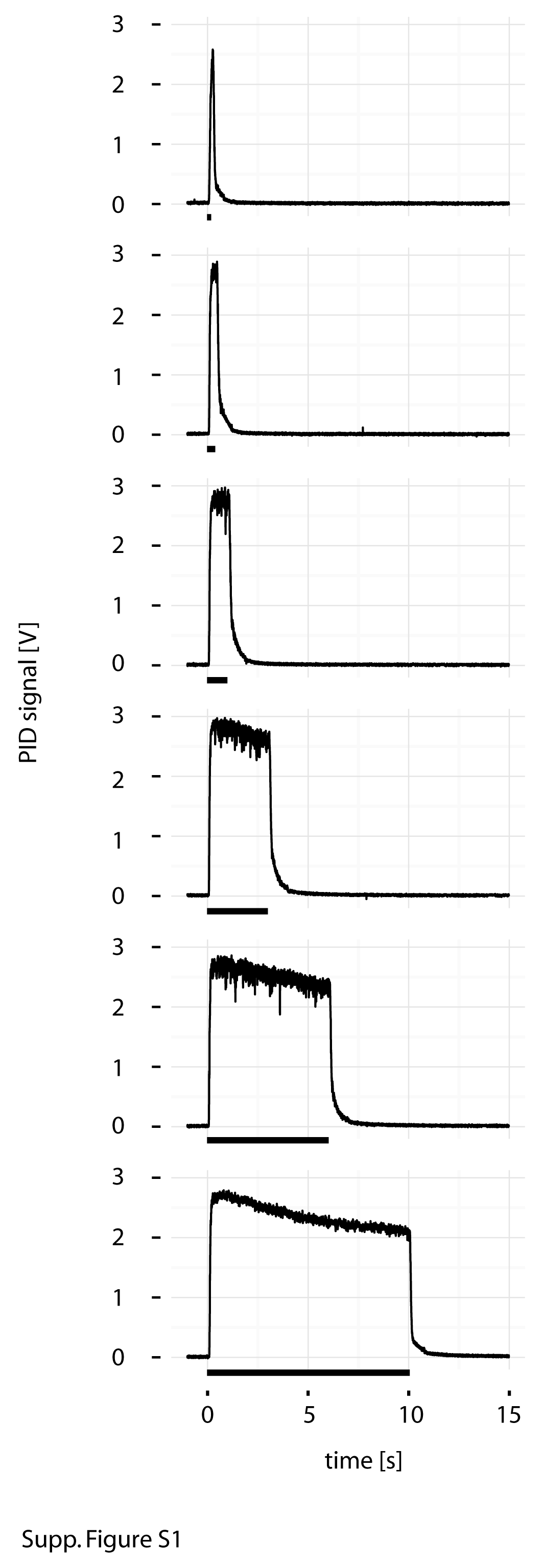

Supplement: FIGURE S1 — The olfactory stimulator produced odorant pulses, with steep odorant on- and off-sets. Traces show photoionization detector signals (PID, Model 200a, Aurora Scientific Inc.) during stimulation with the tracer substance ethyl acetate (undiluted, ionization potential 10.01 eV) for different pulse lengths (0.2, 0.4, 1, 3, 6 s in channel 1, and 10 s in channel 2; indicated by the bar below each graph, single measurements). For PID traces to other odorants applied with a similar olfactory stimulator, see (Raiser et al., 2017). [file Image_1.TIF]

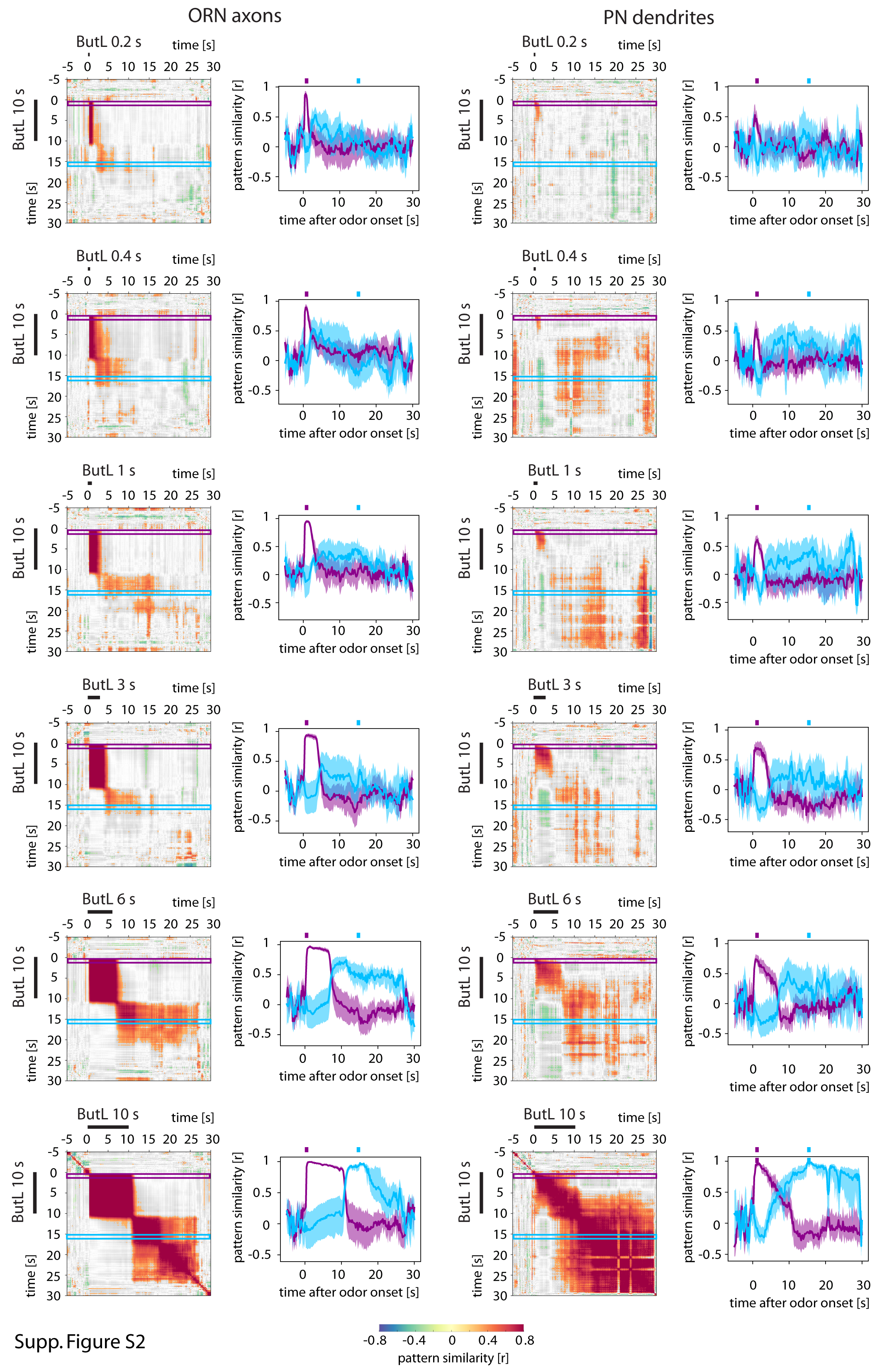

Supplement: FIGURE S2 — Related to Figure 4: Post-odor response patterns of ORN axons and PN dendrites differ from odor response patterns. Corresponding graphs as in Figures 3A,B, for cross correlations between stimuli of increasing length (abscissa, from top to bottom), with a stimulus of 10 s length (ordinate, in each graph). [file Image_2.TIF]

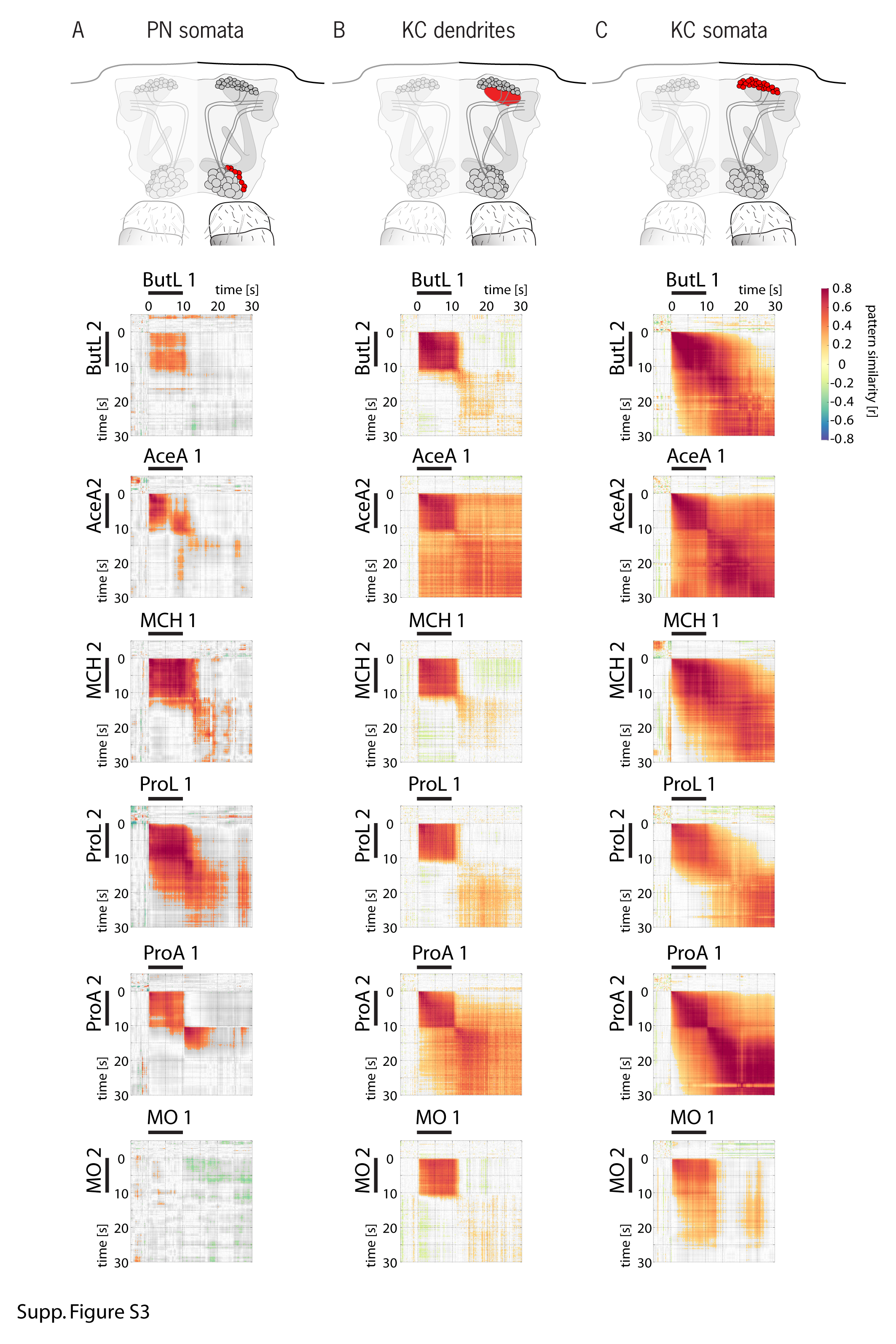

Supplement: FIGURE S3 — Related to Figure 6: Odor and post-odor responses evolve differently in different brain areas. Corresponding graphs as in Figure 6 for cross correlations between responses to repeated presentation of the same odorant, in PN somata, KC dendrites and KC somata [columns (A–C), respectively]. See methods for odorant abbreviations. [file Image_3.TIF]
